# Hematological and biochemical parameters for Chinese rhesus macaque

**DOI:** 10.1371/journal.pone.0222338

**Published:** 2019-09-17

**Authors:** Wenhai Yu, Xianhui Hao, Fengmei Yang, Jin Ma, Yuan Zhao, Yanyan Li, Junbin Wang, Hongjie Xu, Lixiong Chen, Quan Liu, Suqin Duan, Yaping Yang, Fen Huang, Zhanlong He

**Affiliations:** 1 Institute of Medical Biology, Chinese Academy of Medical Sciences and Peking Union Medical College, Kunming, PR China; 2 Medical Faculty, Kunming University of Science and Technology, Kunming, PR China; University of California Irvine, UNITED STATES

## Abstract

Rhesus macaque is an important animal model in biomedical research, especially human disease, developmental, translational, and pre-clinical research. Blood physiological and biochemical parameters are important markers for physiology, pathology, and toxicology research. However, these parameters have not been systematically reported for Chinese rhesus macaques. To characterize the reference for these parameters, this study collected 1805 Chinese rhesus macaques living in Southwestern China. A total of 24 blood physiological indexes and 27 biochemical parameters were determined. Sex and age were found to affect these parameters. In conclusion, a comprehensive and systematic reference of hematological and biochemical parameters for Chinese rhesus macaque was established in this work on the basis of a large cohort. Such reference will benefit biomedical research employing rhesus macaques as animal models.

## Introduction

Nonhuman primates (NHPs), the closest animal models to humans in terms of genetics, physiology, and behavior, play a major role in current biomedical research [1, 2]. The five commonly used NHP [3] species in biomedical research are rhesus macaque (*Macaca mulatta*) [4–6], cynomolgus macaque (*Macaca fascicularis*) [7, 8], African green monkey (*Chlorocebus aethiops sabaeus*) [9], baboon (*Papio anubis*) [10], and marmoset (*Callithrix jacchus*) [11]. Rhesus macaques share nearly 98% of their genetic homology with humans [12], and their similarities include morphology, reproductive physiological characteristics, and biochemical metabolism. They are recognized as the best and sometimes the only available experimental animals for biomedical or translational research in pharmacology and toxicology, oncology, cardiovascular disease, reproductive medicine, zoonotic transmission, and pre-clinical studies [1, 13–17]. As the most important NHP animal model, rhesus macaques have been widely studied, and their whole-genome sequences, transcriptomes, major histocompatibility complex, and cytochrome P40 genes have been effectively distinguished.

As important markers for physiology, pathology, and toxicology research, the blood physiological and biochemical parameters of rhesus macaques have not been characterized comprehensively. These markers directly reflect physical health status and are useful for clinical diagnosis [18, 19]. Although the hematological and biochemical parameters of *Macaca fascicularis* [20, 21], Sulawesi macaques [22], and Tonkean macaques [23] have been reported, those of Chinese rhesus macaques have been rarely investigated. Chen et al. reported the routine chemistry and hematology parameters of Chinese rhesus macaques (3–5 years old, n = 36). However, these parameters cannot accurately reflect their physical health status because the study employed a small sample size, a limited age range, and incomplete indicators [20, 23–26]. Moreover, these parameters vary in species, age, gender, environment, and pathogen infection [13, 27, 28]. Thus, a reference of blood physiological and biochemical parameters must be established for rhesus macaques, which are the most important animal models for biomedical research.

Southwestern China is a major breeding base for rhesus macaques and has a unique geographical location, thereby providing good living conditions for these animals. In this study, 1805 Chinese rhesus monkeys living in Southwestern China (1049 females and 756 males) were collected and characterized to establish an accurate reference of their hematological and biochemical parameters. A total of 24 hematological indexes and 27 biochemical parameters were measured, and the effects of sex and age were analyzed. The obtained sex- and age-based hematological and biochemical parameters are useful indicators when using rhesus macaques as an animal model.

## Materials and methods

### Ethics statement

The protocol of animal experimentation was approved by the Committee of Laboratory Animal Welfare and Ethics of Institute of Medical Biology, Chinese Academy of Medical Sciences and Peking Union Medical College.

### Animal care

All procedures were carried out under ketamine anesthesia by trained personnel under the supervision of veterinary staff. All efforts were made to ameliorate the welfare of the animals and minimize their suffering in accordance with the recommendations cited in “Weatherall report for the use of non-human primates.” The monkeys were housed individually in stainless steel cages measuring 8 m × 3 m × 3 m (L×W×H) in an animal room controlled at 10°C–25°C and 50% ± 10% relative humidity with fresh air and a 12:12 h light:dark cycle [20, 29]. They were fed with complete formula food, including corn, wheat, fish meal, bean meal, milk, sugar, and fat powder, which were produced under license number of SCXK (Yunnan) K2015-0004. They were provided with tap water and supplemented with various fresh fruits (apple and banana) and vegetables (cabbage, tomato, and carrot). The rhesus macaque farm is located in Yunnan province (longitude: 102°36′ and latitude: 25°3′) at 2172 m above sea level and experiences an annual average temperature of 15°C. Toys or enrichment was provided to the study animals. At the end of the study, the animals were retained for future research.

### Animals and experimental design

A total of 1805 healthy rhesus macaques (1049 females and 756 males) were randomly selected and obtained from the Institute of Medical Biology, Chinese Academy of Medical Sciences and Peking Union Medical College. The experimental animal production license was SCXK (Yunnan) K2015-0004. Before the experiment, the health status of the monkeys was determined on the basis of history, general health, and appearance. The animals were not specific pathogen free as they were infected with other common subclinical viral pathogens, including rhesus cytomegalovirus, simian foamy virus, rhesus monkey rhadinovirus, type D simian retrovirus, and simian T-lymphotropic virus, but they were negative for *Mycobacterium tuberculosis*, S*almonella* Typhi, *Shigella dysenteriae*,and herpes B virus. The monkeys were divided into six groups according to age: infants group, 0–1 years old (n = 409, 247 females and 162 males); juvenile group, 1–3 years old (n = 369, 235 females and 134 males); young adults group, 4–6 years old (n = 458, 214 females and 244 males); adults group, 7–12 years old (n = 411, 283 females and 128 males); middle-aged group, 13–17 years old (n = 124, 63 females and 61 males); and elderly group, ≥18 years old (n = 34, 7 females and 27 males).

### Blood sample collection

Rhesus macaques were anesthetized by ketamine, whose blood samples were collected by a trained veterinarian after fasting about 12 hours. Aliquots (2 mL) were stored in plastic tubes without anti-coagulants for biochemical analysis. Blood samples were allowed to clot at room temperature for 45 min. The serum was separated by centrifugation at 1600 g for 15 min and analyzed for biochemical parameters immediately. The other 2 mL aliquots were individually transferred into ethylene diamine tetraacetic acid potassium (EDTA-K2) tubes for hematological analysis [20].

### Hematological analysis

Whole blood was collected in EDTA-K2 tubes for analysis of 24 hematological parameters using an automatic hematological analyzer (XT2000, SYSMEX, Japan). Hematological parameters studied include number of white blood cell (WBC), number of red blood cell (RBC), hemoglobin (HGB), hematocrit (HCT), platelets (PLT), mean platelet volume (MPV), plateletcrit (PCT), mean corpuscular volume (MCV), mean corpuscular hemoglobin (MCH), mean corpuscular hemoglobin concentration (MCHC), neutrophil percentage (NEUT%), monocyte percentage (MONO%), eosinophil percentage (EO%), basophilic leukocyte percentage (BASO%), lymphocyte percentage (LYMPH%), neutrophil (NEUT#), lymphocyte (LYMPH#), monocyte (MONO#), eosinophil (EO#), basophil (BASO#), red blood cell volume distribution width-SD (RDW-SD), red blood cell volume distribution width-CV (RDW-CV), plate volume distribution width (PDW), and platelet large cell ratio (P-LCR).

### Serum biochemistry analysis

The following 27 biochemistry parameters were measured using a serum chemistry analyzer (BS-200, Mindray, China): alanine aminotransferase (ALT), aspartate aminotransferase (AST), alkaline phosphatase (ALP), total protein (TP), albumin (ALB), globulin (GLB), albumin:globulin ratio (A/G), total bilirubin (T-BIL), direct bilirubin (D-BIL), indirect bilirubin (I-BIL), gamma glutamyl transferase (r-GT), uric acid (UA), blood urea nitrogen (UREA), creatinine (CREA), glucose (GLU), total cholesterol (TC), triglycerides (TG), high-density lipoprotein (HDL-C), low-density lipoprotein (LDL-C), complement C3 (C3), complement C4 (C4), immunoglobulin A (IgA), immunoglobulin G (IgG), immunoglobulin M (IgM), calcium (Ca), magnesium (Mg), and iron (Fe).

### Statistical analysis

All data were presented as means ± standard errors. A two-way unbalanced analysis of variance (ANOVA) was used to examine the effects of sex, age, and sex–age interaction using SAS software (v6.12). The difference between male and female in each age group was analyzed by post-hoc pair-wise comparisons. P value < 0.05 was considered statistically significant.

## Results

### Hematological parameters of rhesus macaques

**Fig 1:**
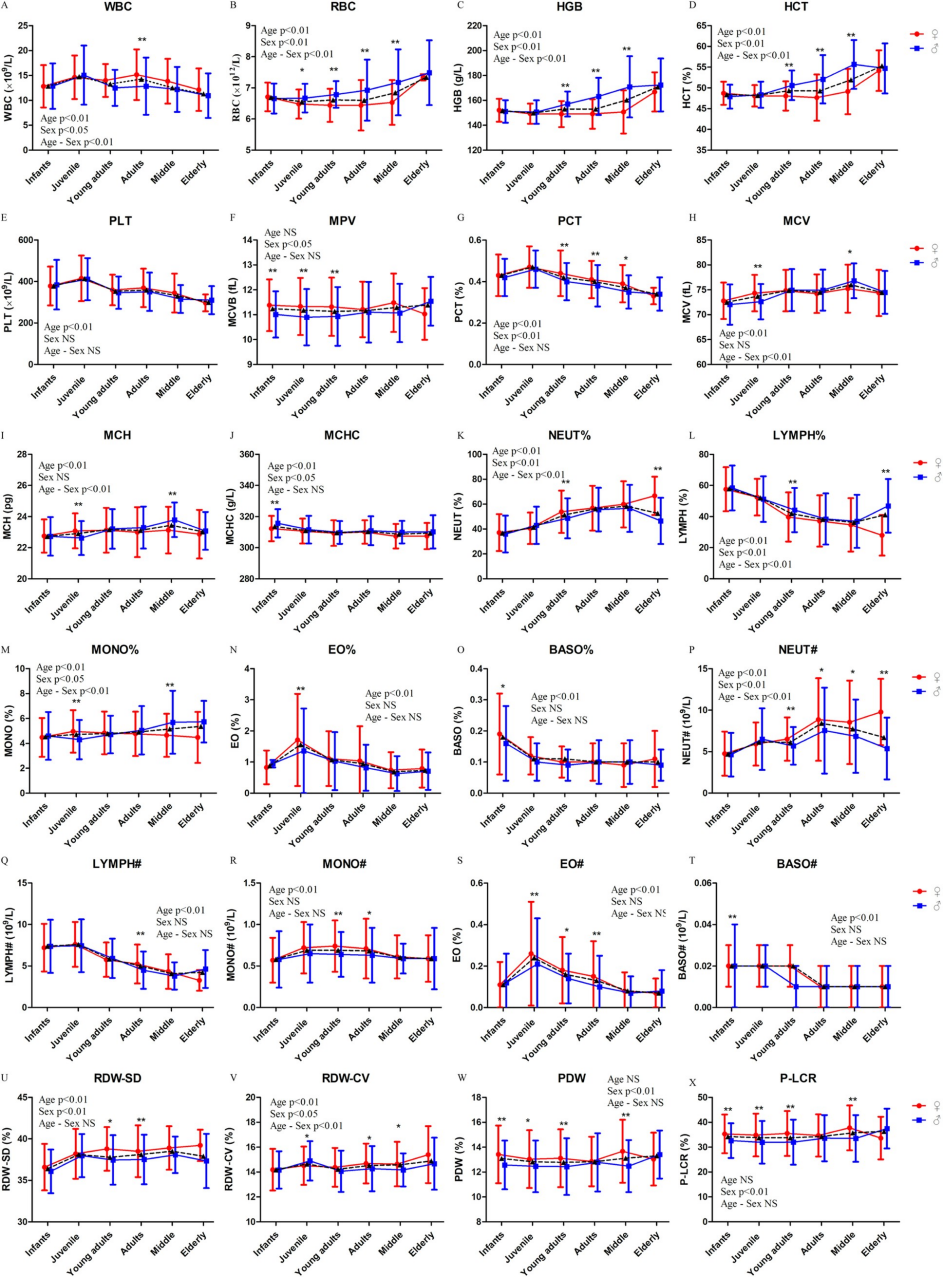
Hematological parameters of rhesus macaques. Represents the mean values of males and females. Two-way ANOVA was used to evaluate the effects of sex, age, and sex–age interaction. The difference between male and female in each age group was analyzed by post-hoc pair-wise comparisons. (*, P<0.05; **, p<0.01).

A total of 24 hematological parameters from 1805 healthy rhesus macaques (1049 females and 756 males) were analyzed. Interestingly, age exerted a significant effect on most hematological parameters, except MPV, PDW, and P-LCR (Table 1, Fig 1 and S1 Table). Sex and gender affect the pathophysiology, incidence, prevalence, symptoms and signs, and course and response to therapy of many diseases. The differences in the effects of sex are important in physiology and disease as they represent gender-related biological factors that may lead to enhanced prevention and therapy [30]. Significant differences were observed between females and males across all hematological parameters, except PLT (Table 1, Fig 1 and S1 Table). Age–sex interaction showed significant effects on WBC, RBC, HGB, HCT, MCV, MCH, NEUT%, LYMP%, MONO%, NEUT#, and RDW-CV (Table 1).

**Table 1:** Summary of effects of age and sex on hematological parameters.

	Parameter	Age	Sex	Interaction
1	WBC	F(5, 1805) = 13.06, *P*<0.01	F(1, 1805) = 4.08, *P*<0.05	F(5, 1805) = 3.38, *P*<0.01
2	RBC	F(5, 1805) = 11.91, *P*<0.01	F(1, 1805) = 30.11, *P*<0.01	F(5, 1805) = 8.44, *P*<0.01
3	HGB	F(5, 1805) = 19.69, *P*<0.01	F(1, 1805) = 74.25, *P*<0.01	F(5, 1805) = 16.47, *P*<0.01
4	HCT	F(5, 1805) = 24.52, *P*<0.01	F(1, 1805) = 35.32, *P*<0.01	F(5, 1805) = 17.65, *P*<0.01
5	PLT	F(5,1805) = 19.02, *P*<0.01	F(1, 1805) = 1.46, NS	F(5, 1805) = 1.41, NS
6	MPV	F(5, 1805) = 0.66, NS	F(1, 1805) = 5.05, *P*<0.05	F(5, 1805) = 1.63, NS
7	PCT	F(5,1805) = 29.78, *P*<0.01	F(1, 1805) = 8.11, *P*<0.01	F(5, 1805) = 1.86, NS
8	MCV	F(5, 1805) = 26.79, *P*<0.01	F(1, 1805) = 0.00, NS	F(5, 1805) = 5.15, *P*<0.01
9	MCH	F(5, 1805) = 8.96, *P*<0.01	F(1, 1805) = 1.73, NS	F(5, 1805) = 4.29, *P*<0.01
10	MCHC	F(5,1805) = 9.97, *P*<0.01	F(1, 1805) = 5.44, *P*<0.05	F(5, 1805) = 0.80, NS
11	NEUT%	F(5, 1805) = 84.08, *P*<0.01	F(1, 1805) = 14.42, *P*<0.01	F(5, 1805) = 4.06, *P*<0.01
12	LYMPH%	F(5, 1805) = 98.09, *P*<0.01	F(1, 1805) = 15.13, *P*<0.01	F(5, 1805) = 3.83, *P*<0.01
13	MONO%	F(5, 1805) = 3.84, *P*<0.01	F(1, 1805) = 5.06, *P*<0.05	F(5, 1805) = 5.51, *P*<0.01
14	EO%	F(5, 1805) = 19.34, *P*<0.01	F(1, 1805) = 2.16, NS	F(5, 1805) = 1.95, NS
15	BASO#	F(5, 1805) = 44.62, *P*<0.01	F(1, 1805) = 1.08, NS	F(5, 1805) = 1.72, NS
16	NEUT#	F(5, 1805) = 40.39, *P*<0.01	F(1, 1805) = 20.38, *P*<0.01	F(5, 1805) = 6.03, *P*<0.01
17	LYMPH#	F(5, 1805) = 62.31, *P*<0.01	F(1, 1805) = 0.04, NS	F(5, 1805) = 2.04, NS
18	MONO#	F(5, 1805) = 9.95, *P*<0.01	F(1, 1805) = 3.09, NS	F(5, 1805) = 2.17, NS
19	EO#	F(5, 1805) = 29.35, *P*<0.01	F(1, 1805) = 3.16, NS	F(5, 1805) = 2.19, NS
20	BASO%	F(5, 1805) = 21.41, *P*<0.01	F(1, 1805) = 2.23, NS	F(5, 1805) = 0.34, NS
21	RDW-SD	F(5, 1805) = 22.16, *P*<0.01	F(1, 1805) = 14.13, *P*<0.01	F(5, 1805) = 0.94, NS
22	RDW-CV	F(5, 1805) = 5.65, *P*<0.01	F(1, 1805) = 5.41, *P*<0.05	F(5, 1805) = 3.30, *P*<0.01
23	PDW	F(5, 1805) = 1.29, NS	F(1, 1805) = 8.52, *P*<0.01	F(5, 1805) = 1.99, NS
24	P-LCR	F(5, 1805) = 1.63, NS	F(1, 1805) = 7.26, *P*<0.01	F(5, 1805) = 1.9, NS

NS: not significant

### Biochemical parameters of rhesus macaques

**Fig 2:**
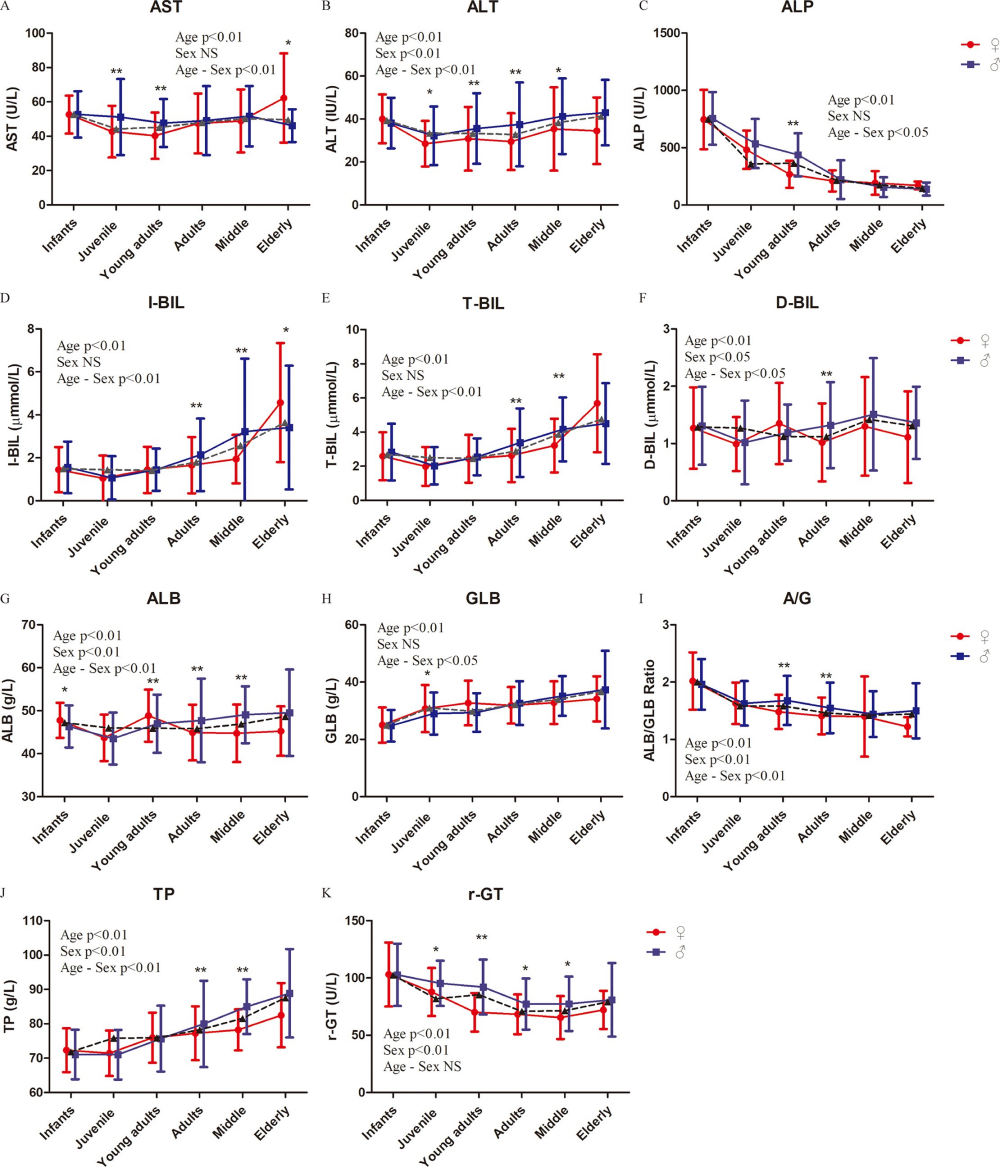
Liver enzyme activities of rhesus macaques. Represents the mean values of males and females. Two-way ANOVA was used to evaluate the effects of sex, age, and sex–age interaction. The difference between male and female in each age group was analyzed by post-hoc pair-wise comparisons. (*, P<0.05; **, p<0.01).

Significant differences were found in the biochemical parameters of all liver enzymes by age group (Fig 2, Table 2 and S2 Table). The changes in the activities of liver enzymes in different age groups were consistent with previous reports on Chinese rhesus macaques [29, 31]. The activities of liver enzymes ALT, A/G, D-BIL, TP, ALB, and rGT were significantly different between females and males (Fig 2, Table 2 and S2 Table). A significant sex-related difference was observed in the AST activity of the juvenile, young adults, and elderly groups, whereas no significant difference was observed in the infants, adults, and middle-aged groups. A significant sex-related difference was observed in the ALT levels of the juvenile to middle-aged groups, whereas no significant difference was observed in the infants and early groups (Fig 2, Table 2 and S2 Table). Significant effects by age–sex interaction were found in all activities of liver enzymes, except rGT (Fig 2 and Table 2).

**Table 2:** Summary of effects of age and sex on biochemical parameters.

	Parameter	Age	Sex	Interaction
1	AST	F(5, 1805) = 10.95, *P*<0.01	F(1, 1805) = 0.25, NS	F(5,1805) = 5.25, *P*<0.01
2	GLB	F(5, 1805) = 61.25, *P*<0.01	F(1, 1805) = 0.81, NS	F(5,1805) = 2.68, *P*<0.05
3	ALT	F(5, 1805) = 14.84, *P*<0.01	F(1, 1805) = 17.67, *P*<0.01	F(5,1805) = 6.73, *P*<0.01
4	I-BIL	F(5, 1805) = 43.18, *P*<0.01	F(1, 1805) = 1.08, NS	F(5,1805) = 6.95, *P*<0.01
5	T-BIL	F(5, 1805) = 36.22, *P*<0.01	F(1, 1805) = 1.45, NS	F(5,1805) = 6.03, *P*<0.01
6	A/G	F(5, 1805) = 77.12, *P*<0.01	F(1, 1805) = 8.06, *P*<0.01	F(5,1805) = 5.52, *P*<0.01
7	D-BIL	F(5, 1805) = 9.89, *P*<0.01	F(1, 1805) = 4.82, *P*<0.05	F(5,1805) = 2.46, *P*<0.05
8	TP	F(5, 1805) = 66.39, *P*<0.01	F(1, 1805) = 11.8, *P*<0.01	F(5,1805) = 6.66, *P*<0.01
9	ALB	F(5, 1805) = 12.79, *P*<0.01	F(1, 1805) = 14.47, *P*<0.01	F(5,1805) = 7.82, *P*<0.01
10	ALP	F(5, 1805) = 74.28, *P*<0.01	F(1, 1805) = 0.19, NS	F(5,1805) = 2.38, *P*<0.05
11	r-GT	F(5, 1805) = 47.86, *P*<0.01	F(1, 1805) = 9.19, *P*<0.01	F(5,1805) = 1.19, NS
12	UA	F(5, 1805) = 16.64, *P*<0.01	F(1, 1805) = 17.04, *P*<0.01	F(5,1805) = 4.96, *P*<0.01
13	UREA	F(5, 1805) = 4.74, *P*<0.01	F(1, 1805) = 0.05, NS	F(5,1805) = 11.25, *P*<0.01
14	CREA	F(5, 1805) = 121.67, *P*<0.01	F(1, 1805) = 101.49, *P*<0.01	F(5,1805) = 31.45, *P*<0.01
15	GLU	F(5, 1805) = 58.82, *P*<0.01	F(1, 1805) = 1.21, NS	F(5,1805) = 1.12, NS
16	TC	F(5, 1805) = 29.31, *P*<0.01	F(1, 1805) = 7.15, *P*<0.01	F(5,1805) = 3.31, *P*<0.01
17	TG	F(5, 1805) = 14.97, *P*<0.01	F(1, 1805) = 0.67, NS	F(5,1805) = 5.69, *P*<0.01
18	HDL-C	F(5, 1805) = 15.74, *P*<0.01	F(1, 1805) = 1.33, *P* = 0.25	F(5,1805) = 14.98, *P*<0.01
19	LDL-C	F(5, 1805) = 0.98, NS	F(1, 1805) = 0.00, NS	F(5,1805) = 0.82, NS
20	C3	F(5, 1805) = 84.21, *P*<0.01	F(1, 1805) = 2.87, NS	F(5,1805) = 5.32, *P*<0.01
21	C4	F(5, 1805) = 6.04, *P*<0.01	F(1, 1805) = 0.54, NS	F(5,1805) = 1.06, NS
22	IgA	F(5, 1805) = 32.13, *P*<0.01	F(1, 1805) = 2.07, NS	F(5,1805) = 0.40, NS
23	IgG	F(5, 1805) = 94.94, *P*<0.01	F(1, 1805) = 1.54, NS	F(5,1805) = 4.11, *P*<0.01
24	IgM	F(5, 1805) = 34.96, *P*<0.01	F(1, 1805) = 0.01, NS	F(5,1805) = 1.70, NS
25	Ca	F(5, 1805) = 12.77, *P*<0.01	F(1, 1805) = 0.01, NS	F(5,1805) = 3.47, *P*<0.01
26	Mg	F(5, 1805) = 14.51, *P*<0.01	F(1, 1805) = 1.40, NS	F(5,1805) = 1.32, NS
27	Fe	F(5, 1805) = 19.84, *P*<0.01	F(1, 1805) = 5.04, *P*<0.05	F(5,1805) = 2.53, *P*<0.05

NS: not significant

**Fig 3:**
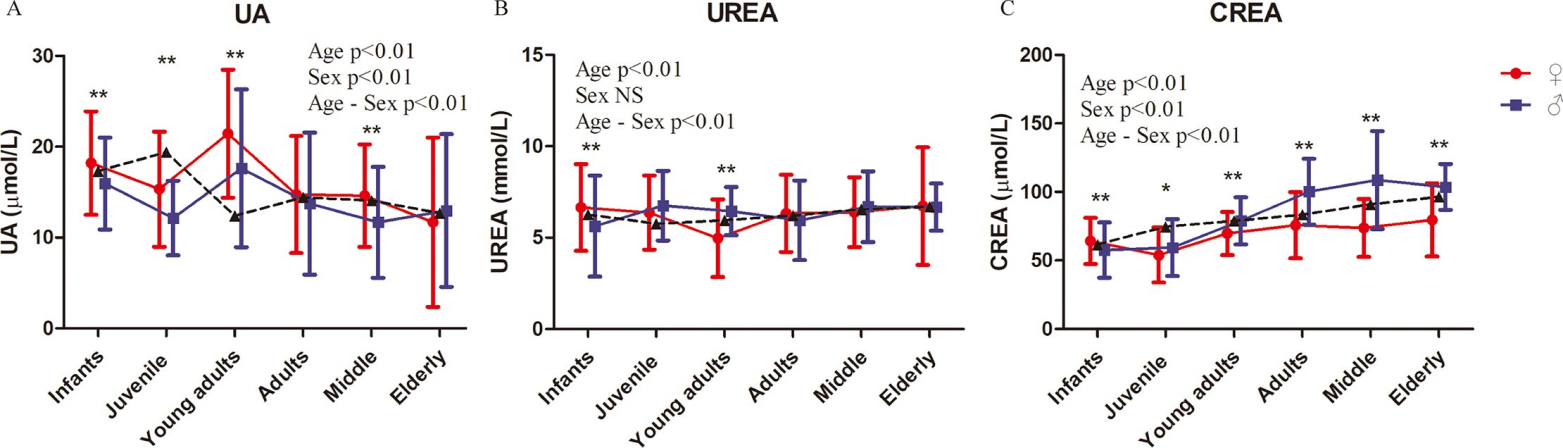
Renal function index of rhesus macaques. Represents the mean values of males and females. Two-way ANOVA was used to evaluate the effects of sex, age, and sex–age interaction. The difference between male and female in each age group was analyzed by post-hoc pair-wise comparisons. (*, P<0.05; **, p<0.01).

The risk of chronic kidney disease was reported to be higher in low birth weight men than in low birth weight women [32, 33]. Similar to humans, male and female rhesus macaques showed significant differences in renal function parameters, including UA and CREA. Age-related differences in the decline in renal function and injury of male and female offspring have been reported in rats [34, 35]. In the current work, the renal function of rhesus macaques was also significantly affected by age (Fig 3, Table 2 and S3 Table). Significant effects by age–sex interaction were found in all renal function parameters (Fig 3 and Table 2).

**Fig 4:**
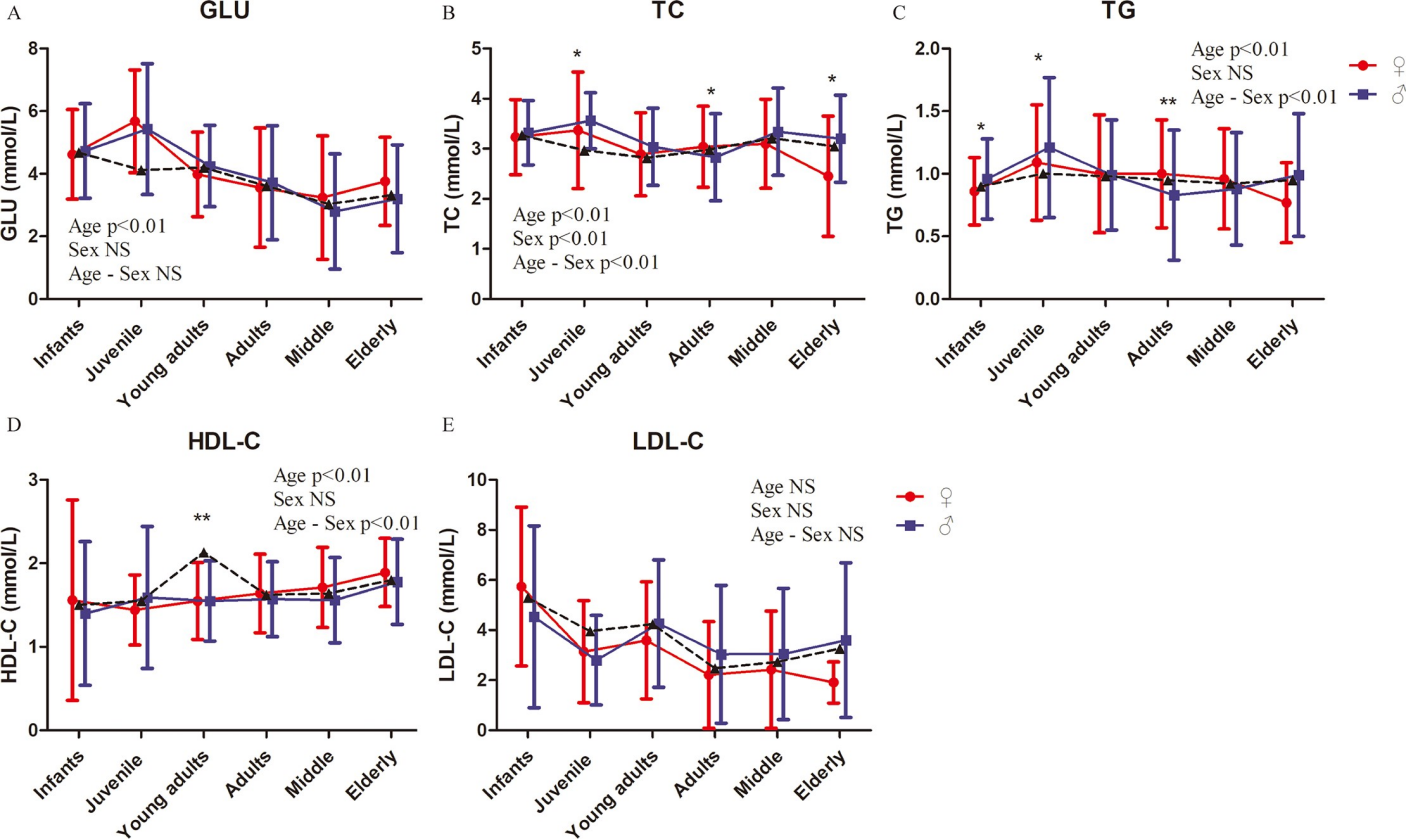
Blood glucose and blood lipid indexes of rhesus macaques. Represents the mean values of males and females. Two-way ANOVA was used to evaluate the effects of sex, age, and sex–age interaction. The difference between male and female in each age group was analyzed by post-hoc pair-wise comparisons. (*, P<0.05; **, p<0.01).

Although glucose homeostasis is regulated differently in males and females [30], GLU and TG of male and female rhesus do not significantly differ, but the opposite is true for TC (Fig 4, Table 2 and S4 Table). Sex disparities have been previously reported for LDL physicochemical properties, with men being characterized as having a higher proportion of sdLDL and greater concentrations of oxLDL than premenopausal women [36–38]. However, no significant sex-related difference was found in HDL-C and LDAL-C in the current work (Fig 4, Table 2 and S4 Table). On the contrary, significant age-related differences in GLU, TC, TG, and HDL-C were found (Fig 4, Table 2 and S4 Table). Significant effects by age–sex interaction were found in TC, TG, and HDL-C (Fig 4 and Table 2).

**Fig 5:**
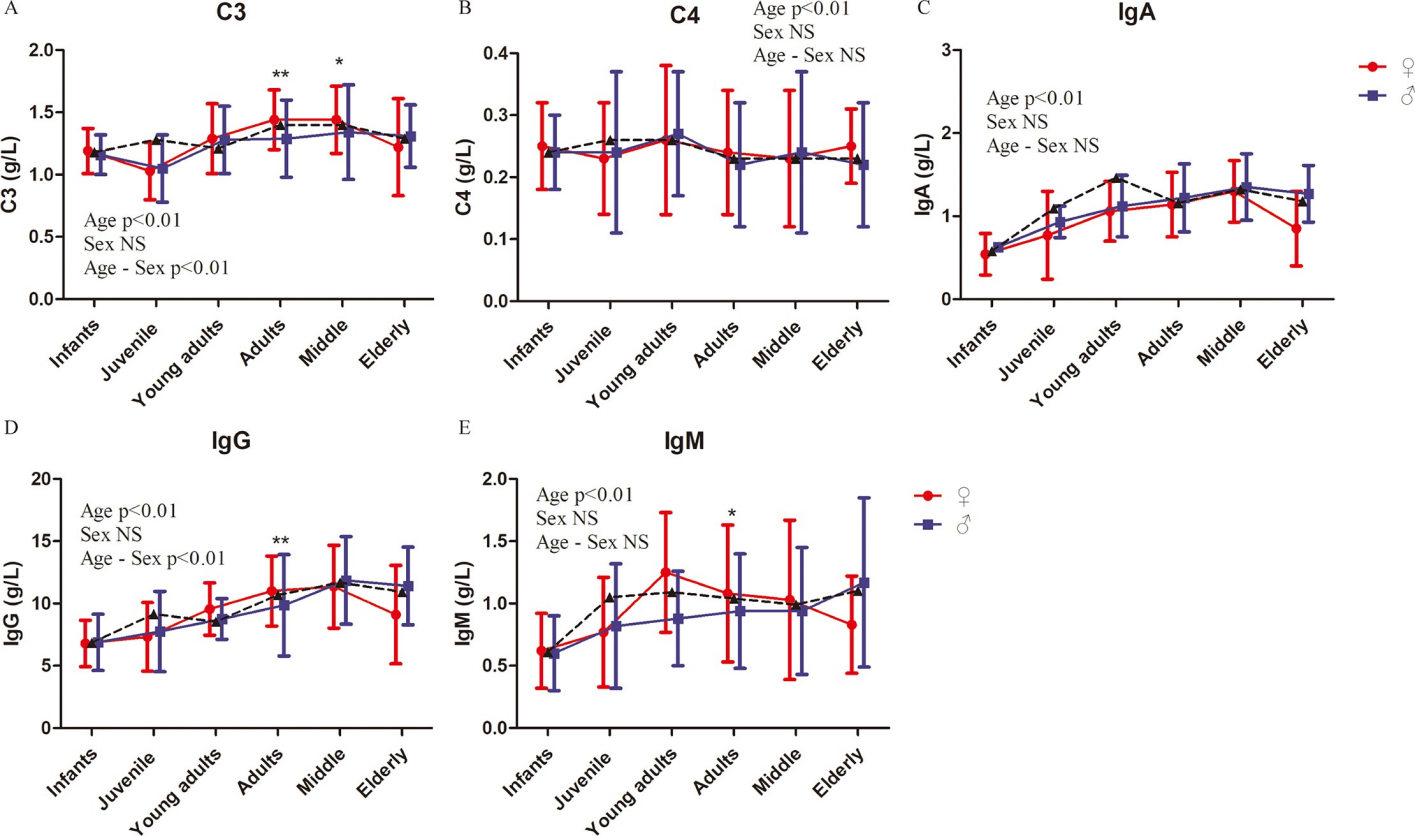
Complement and immunoglobulin index of rhesus macaques. Represents the mean values of males and females. Two-way ANOVA was used to evaluate the effects of sex, age, and sex–age interaction. The difference between male and female in each age group was analyzed by post-hoc pair-wise comparisons. (*, P<0.05; **, p<0.01).

The complement system is an ancient and critical effector mechanism of the innate immune system as it senses, kills, and clears infectious and/or dangerous particles; it also alerts the immune system about the presence of infections and/or danger [39]. It is powerful and is composed of >30 proteins found in circulation and tissues [39]. It is the effector of immune cytolysis and other biologic functions, including anaphylaxis [40], phagocytosis [41], opsonization, and hemolysis [42]. Its background in rhesus macaques is important, especially when these animals are used as animal models. Despite the significant age-related differences in the levels of C3 and C4, no significant sex-related difference was observed (Fig 5, Table 2 and S5 Table). Significant effects by age–sex interaction were found in the level of C3 but not in the level of C4 (Fig 5 and Table 2).

Immunoglobulins (Ig), proteins of animal origin with known antibody activities, are the major components of the humoral immune response system. The H chain comes in five antigenically different types, which serve as the basis for their classification. The five major classes of Igs are IgA, IgD, IgE, IgG, and IgM [43, 44]. Changes in IgG glycosylation drastically alter its function and are related to the age, sex, and disease status of an individual [45, 46]. However, as the most important animal model in biomedical research, rhesus macaques are rarely studied from the aspect of their Igs. In the present study, the Igs of rhesus macaques in a large cohort were investigated. Interestingly, the concentrations of IgA, IgG, and IgM showed a significant age-related difference among the six groups, whereas no significant sex-related difference was observed. Significant effects by age–sex interaction were only found in the concentration of IgG but not in those of IgA and IgM (Fig 5, Table 2 and S5 Table).

**Fig 6:**
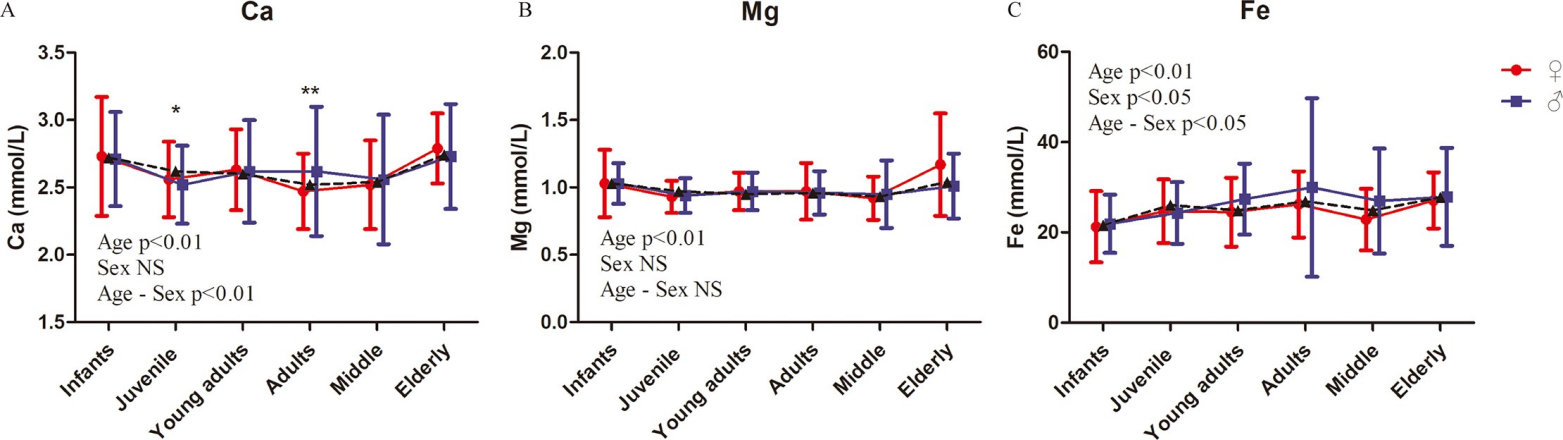
Ion/Electrolyte indexes of rhesus macaques. Represents the mean values of males and females. Two-way ANOVA was used to evaluate the effects of sex, age, and sex–age interaction. The difference between male and female in each age group was analyzed by post-hoc pair-wise comparisons. (*, P<0.05; **, p<0.01).

Ca, Mg, and Fe are important for development. Ca is vital for organisms and constitutes essential components of the skeleton. Its concentration in serum is tightly hormonally regulated [47]. In this study, significant age-related differences were found in the concentrations of Ca, Mg, and Fe of rhesus macaques (Fig 6, Table 2 and S6 Table). Sex-related differences in Ca concentration have been reported in rhesus macaques [48] and vervet monkeys [49]. However, no significant sex-related difference in Ca and Mg and a significant sex-related difference in Fe were found in the male and female rhesus macaques.

## Discussion

NHPs are widely used in studies of human diseases because of their high similarity to humans, and thus, they largely contribute to the development of medicine and other disciplines [4, 15, 50]. NHPs (except chimpanzees) are also valuable animal models in the research on aging diseases, reproductive physiology, behavior, virology, and neurophysiology [5, 51] because of their homology with humans. Chinese rhesus macaques are the major animal models for biotechnology, pharmaceutical, and medical research worldwide, and China has become one of the major breeders and suppliers of rhesus macaques used for biomedical research.

Hematological and biochemical parameters are important indicators in biology and medical research. They are used to judge the health status of animals and thus provide important references in the study of pathology and toxicology and directly and indirectly reflect organ functions [21, 27, 52]. In recent years, as an important animal model, Chinese rhesus macaques have been increasingly used for biomedical research, including disease development, establishment of transgenic animals and stem cells, construction of animal models of diseases, and pre-clinical investigations [53–56]. However, comprehensive and systematic reference ranges of their blood physiological and biochemical parameters have not been established yet.

In this study, blood hematological and biochemical parameters from a large cohort of Chinese rhesus macaques (n = 1805) were analyzed on the basis of gender and age. All age ranges (infants, juvenile, young adults, adults, middle-aged, and elderly) were covered in the study. As the sample size employed was large, the study was able to establish the most suitable reference values per age group. Moreover, some parameters that have never been reported, such as IgG, IgM, and IgA, were evaluated in this study. Therefore, the developed reference ranges of blood hematological and biochemical parameters are comprehensive and accurate. In addition, the effects of the interaction of age and sex on these blood indexes were analyzed. Thus, a differential analysis between females and males or among different age groups must be conducted when using Chinese rhesus macaques as an experimental animal model.

Blood hematological and biochemical parameters varied in different species. Some parameters of Chinese rhesus macaques differed from those of other monkeys. For example, the parameters WBC and RBC of Chinese rhesus macaques are consistent with those of Japanese monkeys [57] and cynomolgus monkeys [58], but PLT in Chinese rhesus macaques is higher than that in *Macaca mulatta* [31]. In addition, living conditions and geographical origins contribute to the differences. For example, the levels of WBC, RBC, HGB, HCT, and PLT in rhesus macaques imported from China to Japan [57] are lower than those in rhesus macaques living in Southwestern China.

## Supporting information

S1 Table Hematological parameters of rhesus macaques. (DOCX)

S2 Table Liver enzymes activities of rhesus macaques. (DOCX)

S3 Table Renal function index of rhesus macaques. (DOCX)

S4 Table Blood glucose and blood lipid index of rhesus macaques. (DOCX)

S5 Table Immunoglobulin and complement index of rhesus macaques. (DOCX)

S6 Table Ion/Electrolyte indexes of rhesus macaques. (DOCX)
