# Supplementary material for: Hematological and biochemical parameters for Chinese rhesus macaque
Source: PLoS One. 2019 Sep 17;14(9):e0222338. doi: 10.1371/journal.pone.0222338 (PMC6748566; doi:10.1371/journal.pone.0222338)
Supplement: S1 Table — (DOCX) [file pone.0222338.s001.docx]

**S1 Table. Hematological parameters of rhesus macaques**

| Parameter  (Unit) | Sex | Infants | Juvenile | Young adults | Adults | Middle | Elderly | P values |
| --- | --- | --- | --- | --- | --- | --- | --- | --- |
| WBC | ♀ | 12.83 ± 4.25 | 14.64 ± 4.38 | 14.00 ± 3.30 | 15.16 ± 5.08 | 13.84 ± 4.50 | 12.14 ± 4.26 |  |
| (10^9^/L) | ♂ | 12.86 ± 4.56 | 15.07 ± 5.94 | 12.52 ± 3.61 | 12.85 ± 5.74 | 12.20 ± 4.52 | 10.95 ± 4.48 |  |
|  |  | P=0.95 | P=0.43 | P=0.72 | P< 0.01 | P=0.13 | P=0.52 |  |
|  | ♀+♂ | 12.84 ± 4.37 | 14.76 ± 4.86 | 13.28 ± 3.53 | 14.26 ± 5.46 | 12.52 ± 4.70 | 11.31 ± 4.38 | P< 0.01 |
| RBC | ♀ | 6.71 ± 0.46 | 6.48 ± 0.47 | 6.44 ± 0.53 | 6.44 ± 0.81 | 6.53 ± 0.72 | 7.34 ± 0.10 |  |
| (10^12^/L) | ♂ | 6.66 ± 0.48 | 6.67 ± 0.46 | 6.79 ± 0.43 | 6.93 ± 0.98 | 7.18 ± 1.06 | 7.49 ± 1.04 |  |
|  |  | P=0.52 | P=0.013 | P< 0.01 | P< 0.01 | P< 0.01 | P=0.54 |  |
|  | ♀+♂ | 6.69 ± 0.47 | 6.55 ± 0.48 | 6.61 ± 0.52 | 6.63 ± 0.91 | 6.84 ± 0.95 | 7.34 ± 0.10 | P< 0.01 |
| HGB | ♀ | 152.10 ± 9.20 | 149.30 ± 8.07 | 149.00 ± 10.48 | 149.10 ± 11.88 | 150.70 ± 17.32 | 166.80 ± 15.73 |  |
| (g/L) | ♂ | 151.10 ± 9.03 | 150.60 ± 9.45 | 157.10 ± 10.03 | 163.20 ± 14.90 | 170.90 ± 24.59 | 172.40 ± 21.26 |  |
|  |  | P=0.49 | P=0.43 | P< 0.01 | P< 0.01 | P< 0.01 | P=0.32 |  |
|  | ♀+♂ | 151.70 ± 9.13 | 149.80 ± 8.61 | 153.0 ± 11.02 | 153.00 ± 20.71 | 160.30 ± 23.31 | 170.70 ± 19.67 | P< 0.01 |
| HCT | ♀ | 48.71 ± 2.77 | 48.09 ± 2.57 | 48.06 ± 3.53 | 47.66 ± 5.57 | 49.13 ± 5.46 | 54.16 ± 4.91 |  |
| (%) | ♂ | 47.88 ± 2.87 | 48.36 ± 3.14 | 50.62 ± 3.56 | 52.09 ± 5.83 | 55.65 ± 5.88 | 54.7 ± 6.04 |  |
|  |  | P= 0.07 | P=0.59 | P< 0.01 | P< 0.01 | P< 0.01 | P=0.38 |  |
|  | ♀+♂ | 48.39 ± 2.84 | 48.18 ± 2.79 | 49.31 ± 3.76 | 49.25 ± 6.49 | 51.93 ± 7.18 | 55.29 ± 6.98 | P< 0.01 |
| PLT | ♀ | 378.20 ± 93.40 | 415.00 ± 110.00 | 359.10 ± 73.66 | 369.30 ± 92.60 | 344.60 ± 93.82 | 296.90 ± 40.73 |  |
| (10^9^/L) | ♂ | 384.70 ± 119.40 | 411.20 ± 101.40 | 346.20 ± 78.02 | 350.50 ± 92.24 | 315.40 ± 67.13 | 310.20 ± 67.42 |  |
|  |  | P=0.31 | P=0.74 | P=0.06 | P= 0.07 | P=0.12 | P=0.93 |  |
|  | ♀+♂ | 378.40 ± 107.80 | 413.60 ± 106.80 | 352.8 ± 76.01 | 358.50 ± 98.08 | 330.70 ± 83.18 | 299.30 ± 70.58 | P< 0.01 |
| MPV | ♀ | 11.38 ± 1.04 | 11.33 ± 1.15 | 11.32 ± 1.17 | 11.21 ± 1.11 | 11.48 ± 1.71 | 11.03 ± 1.03 |  |
| (fL) | ♂ | 11.01 ± 0.93 | 10.90 ± 1.13 | 10.93 ± 1.18 | 11.10 ± 1.22 | 11.07 ± 1.17 | 11.54 ± 0.98 |  |
|  |  | P< 0.01 | P< 0.01 | P< 0.01 | P=0.35 | P=0.052 | P=0.23 |  |
|  | ♀+♂ | 11.24 ± 1.02 | 11.18 ± 1.16 | 11.13 ± 1.19 | 11.16 ± 1.16 | 11.29 ± 1.19 | 11.39 ± 1.01 | P=0.41 |
| PCT | ♀ | 0.43 ± 0.10 | 0.47 ± 0.10 | 0.44 ± 0.11 | 0.41 ± 0.09 | 0.39 ± 0.09 | 0.33 ± 0.04 |  |
| (%) | ♂ | 0.42 ± 0.09 | 0.46 ± 0.09 | 0.40 ± 0.09 | 0.38 ± 0.10 | 0.35 ± 0.08 | 0.34 ± 0.08 |  |
|  |  | P=0.35 | P=0.12 | P< 0.01 | P< 0.01 | P< 0.05 | P=0.69 |  |
|  | ♀+♂ | 0.43 ± 0.10 | 0.47 ± 0.10 | 0.42 ± 0.10 | 0.40 ± 0.10 | 0.37 ± 0.09 | 0.34 ± 0.07 | P< 0.01 |
| MCV | ♀ | 72.79 ± 3.65 | 74.33 ± 3.65 | 74.83 ± 4.16 | 74.22 ± 3.87 | 75.23 ± 4.83 | 74.37 ± 4.63 |  |
| (fL) | ♂ | 72.03 ± 4.04 | 72.61 ± 3.54 | 74.95 ± 4.21 | 74.94 ± 4.12 | 76.81 ± 3.50 | 74.48 ± 4.30 |  |
|  |  | P=0.054 | P< 0.01 | P=0.79 | P=0.09 | P=0.03 | P=0.93 |  |
|  | ♀+♂ | 72.49 ± 3.82 | 73.70 ± 3.70 | 74.88 ± 4.18 | 74.5 ± 3.98 | 75.98 ± 4.31 | 74.45 ± 4.33 | P< 0.01 |
| MCH | ♀ | 22.75 ± 1.07 | 23.08 ± 1.12 | 23.12 ± 1.44 | 23.00 ± 1.60 | 23.13 ± 1.50 | 22.87 ± 1.56 |  |
| (pg) | ♂ | 22.73 ± 1.24 | 22.62 ± 1.09 | 23.21 ± 1.27 | 23.29 ± 1.35 | 23.79 ± 1.11 | 23.08 ± 1.22 |  |
|  |  | P=0.96 | P< 0.01 | P=0.37 | P=0.05 | P<0.01 | P=0.67 |  |
|  | ♀+♂ | 22.73 ± 1.19 | 22.91 ± 1.13 | 23.17 ± 1.36 | 23.11 ± 1.51 | 23.44 ± 1.37 | 23.02 ± 1.31 | P< 0.01 |
| MCHC | ♀ | 312.30 ± 8.14 | 310.70 ± 7.98 | 309.40 ± 8.21 | 310.10 ± 7.54 | 307.40 ± 7.93 | 307.50 ± 8.42 |  |
| (g/L) | ♂ | 315.70 ± 9.06 | 311.60 ± 8.92 | 309.80 ± 7.32 | 310.90 ± 9.24 | 310.00 ± 7.20 | 310.20 ± 10.69 |  |
|  |  | P< 0.01 | P=0.42 | P=0.25 | P=0.44 | P=0.18 | P=0.51 |  |
|  | ♀+♂ | 313.60 ± 8.67 | 311.00 ± 8.34 | 309.6 ± 7.78 | 310.40 ± 13.91 | 308.70 ± 7.67 | 309.40 ± 10.01 | P< 0.01 |
| NEUT% | ♀ | 37.16 ± 14.82 | 40.57 ± 12.65 | 53.87 ± 16.99# | 56.76 ± 17.95 | 59.84 ± 18.55 | 66.67 ± 15.35 |  |
| (%) | ♂ | 35.99 ± 14.82 | 43.00 ± 14.98 | 48.63 ± 16.06 | 55.62 ± 17.47 | 56.63 ± 18.91 | 46.56 ± 18.60 |  |
|  |  | P=0.46 | P=0.17 | P< 0.01 | P=0.51 | P=0.26 | P< 0.01 |  |
|  | ♀+♂ | 36.70 ± 14.81 | 41.46 ± 13.58 | 51.30 ± 16.73 | 56.32 ± 17.75 | 58.32 ± 18.71 | 52.65 ± 19.80 | P< 0.01 |
| LYMPH% | ♀ | 57.60 ± 14.15 | 52.51 ± 11.79 | 39.73 ± 15.83 | 37.18 ± 16.51 | 34.67 ± 17.19 | 27.95 ± 13.08 |  |
| (%) | ♂ | 58.42 ± 14.39 | 51.23 ± 14.65 | 44.20 ± 14.23 | 38.41 ± 16.46 | 36.94 ± 17.08 | 46.91 ± 17.28 |  |
|  |  | P=0.59 | P=0.44 | P< 0.01 | P=0.45 | P=0.40 | P< 0.01 |  |
|  | ♀+♂ | 57.92 ± 14.23 | 52.04 ± 12.91 | 41.92 ± 15.21 | 38.16 ± 16.16 | 36.23 ± 16.82 | 41.16 ± 18.21 | P< 0.01 |
| MONO% | ♀ | 4.48 ± 1.56 | 4.96 ± 1.71 | 4.84 ± 1.71 | 4.767 ± 1.78 | 4.65 ± 1.73 | 4.48 ± 2.05 |  |
| (%) | ♂ | 4.60 ± 1.92 | 4.30 ± 1.58 | 4.71 ± 1.51 | 5.052 ± 1.94 | 5.70 ± 2.53 | 5.74 ± 1.67 |  |
|  |  | P=0.51 | P< 0.01 | P=0.55 | P=0.23 | P< 0.01 | P=0.06 |  |
|  | ♀+♂ | 4.53 ± 1.71 | 4.72 ± 1.69 | 4.78 ± 1.62 | 4.91 ± 1.95 | 5.15 ± 2.20 | 5.36 ± 1.86 | P< 0.01 |
| EO% | ♀ | 0.83 ± 0.84 | 1.71 ± 1.48 | 1.11 ± 0.88 | 1.04 ± 1.11 | 0.74 ± 0.88 | 0.79 ± 0.91 |  |
| (%) | ♂ | 0.95 ± 1.12 | 1.36 ± 1.36 | 1.03 ± 0.93 | 0.82 ± 1.04 | 0.63 ± 0.66 | 0.71 ± 0.80 |  |
|  |  | P=0.24 | P< 0.01 | P=0.33 | P=0.051 | P=0.56 | P=0.84 |  |
|  | ♀+♂ | 0.88 ± 0.96 | 1.56 ± 1.40 | 1.07 ± 0.90 | 0.95 ± 1.09 | 0.69 ± 0.78 | 0.73 ± 0.82 | P< 0.01 |
| BASO% | ♀ | 0.19 ± 0.13 | 0.12 ± 0.06 | 0.10 ± 0.05 | 0.10 ± 0.06 | 0.09 ± 0.07 | 0.11 ± 0.09 |  |
| (%) | ♂ | 0.16 ± 0.12 | 0.10 ± 0.06 | 0.09 ± 0.05 | 0.10 ± 0.07 | 0.10 ± 0.07 | 0.09 ± 0.05 |  |
|  |  | P=0.02 | P= 0.54 | P=0.37 | P=0.17 | P=0.80 | P=0.73 |  |
|  | ♀+♂ | 0.18 ± 0.13 | 0.11 ± 0.06 | 0.11 ± 0.06 | 0.10 ± 0.06 | 0.10 ± 0.07 | 0.10 ± 0.07 | P< 0.01 |
| NEUT# | ♀ | 4.76 ± 2.66 | 5.92 ± 2.60 | 6.52 ± 2.61 | 8.87 ± 4.98 | 8.54 ± 5.03 | 9.78 ± 4.00 |  |
| (10^9^/L) | ♂ | 4.64 ± 2.63 | 6.51 ± 3.72 | 5.70 ± 2.27 | 7.54 ± 5.18 | 6.86 ± 4.42 | 5.38 ± 3.72 |  |
|  |  | P=0.78 | P=0.21 | P< 0.01 | P=0.01 | P=0.04 | P< 0.01 |  |
|  | ♀+♂ | 4.71 ± 2.65 | 6.14 ± 3.06 | 6.12 ± 2.48 | 8.42 ± 5.20 | 7.74 ± 4.81 | 6.71 ± 4.27 | P< 0.01 |
| LYMPH# | ♀ | 7.21 ± 2.87 | 7.61 ± 2.70 | 5.76 ± 2.07 | 5.24 ± 2.34 | 4.33 ± 2.08 | 3.27 ± 1.25 |  |
| (10^9^/L) | ♂ | 7.37 ± 3.20 | 7.44 ± 3.18 | 5.93 ± 2.37 | 4.49 ± 2.25 | 3.80 ± 1.65 | 4.64 ± 2.28 |  |
|  |  | P=0.59 | P=0.82 | P=0.54 | P< 0.01 | P=0.29 | P=0.20 |  |
|  | ♀+♂ | 7.38 ± 3.34 | 7.58 ± 2.96 | 5.85 ± 2.22 | 5.00 ± 2.49 | 4.08 ± 1.90 | 4.22 ± 2.10 | P< 0.01 |
| MONO# | ♀ | 0.57 ± 0.27 | 0.72 ± 0.31 | 0.74 ± 0.31 | 0.71 ± 0.36 | 0.61 ± 0.26 | 0.59 ± 0.28 |  |
| (10^9^/L) | ♂ | 0.58 ± 0.34 | 0.65 ± 0.35 | 0.64 ± 0.27 | 0.63 ± 0.33 | 0.59 ± 0.18 | 0.59 ± 0.37 |  |
|  |  | P=0.56 | P=0.051 | P< 0.01 | P=0.02 | P=0.81 | P=0.98 |  |
|  | ♀+♂ | 0.58 ± 0.31 | 0.69 ± 0.33 | 0.69 ± 0.30 | 0.68 ± 0.35 | 0.60 ± 0.23 | 0.59 ± 0.34 | P< 0.01 |
| EO# | ♀ | 0.11 ± 0.11 | 0.26 ± 0.25 | 0.18 ± 0.16 | 0.15 ± 0.17 | 0.08 ± 0.09 | 0.07 ± 0.07 |  |
| (10^9^/L) | ♂ | 0.12 ± 0.14 | 0.21 ± 0.22 | 0.14 ± 0.12 | 0.10 ± 0.15 | 0.07 ± 0.08 | 0.08 ± 0.10 |  |
|  |  | P=0.42 | P< 0.01 | P=0.017 | P< 0.01 | P=0.56 | P=0.87 |  |
|  | ♀+♂ | 0.11 ± 0.13 | 0.24 ± 0.24 | 0.16 ± 0.14 | 0.13 ± 0.16 | 0.08 ± 0.09 | 0.07 ± 0.09 | P< 0.01 |
| BASO# | ♀ | 0.02 ± 0.03 | 0.02 ± 0.01 | 0.02 ± 0.01 | 0.01 ± 0.01 | 0.01 ± 0.01 | 0.01 ± 0.01 |  |
| (10^9^/L) | ♂ | 0.02 ± 0.02 | 0.02 ± 0.01 | 0.01 ± 0.01 | 0.01 ± 0.01 | 0.01 ± 0.01 | 0.01 ± 0.01 |  |
|  |  | P< 0.01 | P=0.12 | P=0.41 | P=0.97 | P=0.53 | P=0.50 |  |
|  | ♀+♂ | 0.02 ± 0.03 | 0.02 ± 0.01 | 0.02 ± 0.01 | 0.01 ± 0.01 | 0.01 ± 0.01 | 0.01 ± 0.01 | P< 0.01 |
| RDW-SD | ♀ | 36.60 ± 2.80 | 38.20 ± 3.01 | 38.79 ± 2.63 | 38.51 ± 3.12 | 38.90 ± 2.62 | 39.22 ± 1.89 |  |
| (fL) | ♂ | 36.09 ± 2.63 | 37.98 ± 2.59 | 37.46 ± 2.98 | 37.52 ± 2.99 | 38.08 ± 2.20 | 37.34 ± 3.27 |  |
|  |  | P=0.07 | P=0.48 | P= 0.03 | P< 0.01 | P=0.13 | P=0.08 |  |
|  | ♀+♂ | 36.40 ± 2.74 | 38.12 ± 2.86 | 37.78 ± 2.82 | 38.12 ± 3.10 | 38.51 ± 2.46 | 37.91 ± 3.02 | P< 0.01 |
| RDW-CV | ♀ | 14.19 ± 1.67 | 14.51 ± 1.54 | 14.38 ± 1.56 | 14.69 ± 1.60 | 14.64 ± 1.79 | 15.40 ± 2.29 |  |
| (%) | ♂ | 14.17 ± 1.50 | 14.90 ± 1.58 | 14.06 ± 1.66 | 14.27 ± 1.82 | 14.17 ± 1.33 | 14.67 ± 2.09 |  |
|  |  | P=0.87 | P=0.04 | P= 0.06 | P= 0.02 | P=0.013 | P=0.26 |  |
|  | ♀+♂ | 14.18 ± 1.60 | 14.65 ± 1.56 | 14.22 ± 1.62 | 14.52 ± 1.70 | 14.58 ± 2.44 | 14.89 ± 2.14 | P< 0.01 |
| PDW | ♀ | 13.42 ± 2.32 | 13.04 ± 2.32 | 13.11 ± 2.32 | 12.85 ± 1.99 | 13.67 ± 2.53 | 13.04 ± 2.12 |  |
| (%) | ♂ | 12.57 ± 1.96 | 12.46 ± 2.08 | 12.44 ± 2.28 | 12.78 ± 2.34 | 12.48 ± 2.09 | 13.41 ± 1.93 |  |
|  |  | P< 0.01 | P=0.02 | P< 0.01 | P=0.77 | P< 0.01 | P=0.65 |  |
|  | ♀+♂ | 13.09 ± 2.23 | 12.83 ± 2.25 | 12.78 ± 2.32 | 12.83 ± 2.13 | 13.11 ± 2.39 | 13.30 ± 1.96 | p = 0.06 |
| P-LCR | ♀ | 35.32 ± 7.77 | 34.86 ± 8.60 | 35.50 ± 9.04 | 34.70 ± 8.47 | 37.75 ± 9.02 | 33.64 ± 8.58 |  |
| (%) | ♂ | 32.63 ± 6.99 | 31.95 ± 8.55 | 32.00 ± 9.06 | 33.61 ± 9.30 | 33.50 ± 9.39 | 37.48 ± 8.00 |  |
|  |  | P< 0.01 | P< 0.01 | P< 0.01 | P=0.24 | P< 0.01 | P=0.23 |  |
|  | ♀+♂ | 34.26 ± 7.58 | 33.79 ± 8.68 | 33.79 ± 9.20 | 34.28 ± 8.81 | 35.73 ± 9.40 | 36.32 ± 8.24 | p = 0.19 |
