# Supplementary material for: Hematological and biochemical parameters for Chinese rhesus macaque
Source: PLoS One. 2019 Sep 17;14(9):e0222338. doi: 10.1371/journal.pone.0222338 (PMC6748566; doi:10.1371/journal.pone.0222338)
Supplement: S2 Table — (DOCX) [file pone.0222338.s002.docx]

**S2 Table.** **Liver enzymes activities of rhesus macaques.**

| Parameter  (Unit) | Sex | Infants | Juvenile | Young adults | Adults | Middle | Elderly | P values |
| --- | --- | --- | --- | --- | --- | --- | --- | --- |
| AST  (U/L) | ♀ | 52.59 ± 11.06 | 42.67 ± 15.06 | 40.28 ± 13.47 | 47.44 ± 17.42 | 48.84 ± 18.33 | 62.24 ± 25.97 |  |
|  | ♂ | 52.71 ± 13.53 | 51.16 ± 22.16 | 47.66 ± 13.96 | 49.1 ± 20.02 | 51.67 ± 17.52 | 46.2 ± 9.537 |  |
|  |  | P=0.94 | P< 0.01 | P< 0.01 | P=0.34 | P=0.33 | P=0.02 |  |
|  | ♀+♂ | 52.64 ± 12.08 | 44.21 ± 14.21 | 45.13 ± 16.43 | 47.96 ± 18.26 | 50.23 ± 17.92 | 49.51 ± 15.41 | P< 0.01 |
| GLB  (g/L) | ♀ | 25.04 ± 6.15 | 30.73 ± 8.22 | 32.73 ± 7.78 | 31.89 ± 6.36 | 32.84 ± 7.44 | 34.14 ± 7.86 |  |
|  | ♂ | 24.75 ± 5.52 | 29.03 ± 7.34 | 29.40 ± 6.74 | 32.65 ± 7.65 | 35.15 ± 6.91 | 37.37 ± 13.55 |  |
|  |  | P=0.68 | P=0.02 | P=0.06 | P=0.30 | P=0.06 | P=0.27 |  |
|  | ♀+♂ | 24.92 ± 5.90 | 30.95 ± 7.44 | 29.84 ± 6.19 | 32.12 ± 6.79 | 33.98 ± 7.25 | 36.71 ± 12.56 | P< 0.01 |
| ALT | ♀ | 40.06 ± 11.39 | 28.53 ± 10.64 | 30.82 ± 14.83 | 29.47 ± 13.23 | 35.38 ± 19.41 | 34.51 ± 15.5 |  |
| (U/L) | ♂ | 38.09 ± 11.79 | 32.2 ± 13.63 | 35.60 ± 16.40 | 37.52 ± 19.49 | 41.27 ± 17.6 | 43.01 ± 15.23 |  |
|  |  | P=0.20 | P=0.03 | P< 0.01 | P< 0.01 | P=0.03 | P=0.19 |  |
|  | ♀+♂ | 39.28 ± 11.57 | 33.37 ± 15.85 | 33.24 ± 16.35 | 32.8 ± 19.06 | 38.28 ± 18.71 | 41.26 ± 15.45 | P< 0.01 |
| I-BIL | ♀ | 1.45 ± 1.05 | 1.05 ± 1.06 | 1.44 ± 1.08 | 1.66 ± 1.31 | 1.94 ± 1.13 | 4.57 ± 2.77 |  |
| (μmol/L) | ♂ | 1.56 ± 1.20 | 1.07 ± 1.01 | 1.45 ± 0.98 | 2.14 ± 1.69 | 3.22 ± 3.40 | 3.41 ± 2.88 |  |
|  |  | P=0.42 | P=0.91 | P=0.84 | P< 0.01 | P< 0.01 | P=0.04 |  |
|  | ♀+♂ | 1.49 ± 1.115 | 1.44 ± 1.03 | 1.42 ± 1.03 | 1.80 ± 1.45 | 2.57 ± 2.59 | 3.65 ± 2.86 | P< 0.01 |
| T-BIL | ♀ | 2.59 ± 1.40 | 1.99 ± 1.14 | 2.44 ± 1.40 | 2.63 ± 1.56 | 3.21 ± 1.57 | 5.69 ± 2.87 |  |
| (μmol/L) | ♂ | 2.83 ± 1.66 | 2.03 ± 1.09 | 2.55 ± 1.08 | 3.38 ± 2.01 | 4.16 ± 1.87 | 4.50 ± 2.36 |  |
|  |  | P=0.16 | P=0.84 | P=0.36 | P< 0.01 | P< 0.01 | P=0.10 |  |
|  | ♀+♂ | 2.68 ± 1.51 | 2.50 ± 1.24 | 2.45 ± 1.33 | 2.89 ± 1.86 | 3.88 ± 2.88 | 4.75 ± 2.47 | P< 0.01 |
| A/G | ♀ | 2.02 ± 0.50 | 1.63 ± 0.36 | 1.48 ± 0.30 | 1.41 ± 0.32 | 1.40 ± 0.70 | 1.22 ± 0.166 |  |
|  | ♂ | 1.96 ± 0.44 | 1.63 ± 0.39 | 1.68 ± 0.43 | 1.55 ± 0.44 | 1.44 ± 0.40 | 1.50 ± 0.48 |  |
|  |  | P=.0.13 | P=0.96 | P< 0.01 | P< 0.01 | P=0.54 | P=0.12 |  |
|  | ♀+♂ | 2.00 ± 0.477 | 1.58 ± 0.39 | 1.58 ± 0.40 | 1.46 ± 0.37 | 1.42 ± 0.57 | 1.44 ± 0.44 | P< 0.01 |
| D-BIL | ♀ | 1.27 ± 0.71 | 0.99 ± 0.47 | 1.35 ± 0.71 | 1.02 ± 0.68 | 1.30 ± 0.86 | 1.11 ± 0.80 |  |
| (μmol/L) | ♂ | 1.31 ± 0.68 | 1.02 ± 0.73 | 1.19 ± 0.49 | 1.32 ± 0.75 | 1.51 ± 0.98 | 1.36 ± 0.63 |  |
|  |  | P=0.57 | P=0.69 | P= 0.77 | P< 0.01 | P=0.15 | P=0.40 |  |
|  | ♀+♂ | 1.29 ± 0.70 | 1.27 ± 0.61 | 1.12 ± 0.72 | 1.12 ± 0.72 | 1.42 ± 0.92 | 1.31 ± 0.67 | P< 0.01 |
| TP | ♀ | 72.31 ± 6.38 | 71.42 ± 6.60 | 75.94 ± 7.28 | 77.24 ± 7.84 | 78.24 ± 5.95 | 82.49 ± 9.35 |  |
| (g/L) | ♂ | 71.07 ± 7.22 | 71.01 ± 7.22 | 75.68 ± 9.57 | 79.96 ± 12.55 | 84.98 ± 7.97 | 88.87 ± 12.87 |  |
|  |  | P=0.13 | P=0.65 | P=0.90 | P < 0.01 | P < 0.01 | P=0.06 |  |
|  | ♀+♂ | 71.81 ± 6.72 | 75.80 ± 8.57 | 75.91 ± 8.79 | 78.23 ± 8.9 | 81.56 ± 7.76 | 87.56 ± 12.38 | P< 0.01 |
| ALB | ♀ | 47.78 ± 4.08 | 43.71 ± 5.42 | 48.85 ± 6.07 | 44.93 ± 6.47 | 44.75 ± 6.71 | 45.26 ± 5.743 |  |
| (g/L) | ♂ | 46.35 ± 4.91 | 43.53 ± 6.05 | 46.98 ± 6.76 | 47.75 ± 9.71 | 49.07 ± 6.61 | 49.55 ± 10.08 |  |
|  |  | P= 0.02 | P=0.79 | P< 0.01 | P< 0.01 | P< 0.01 | P=0.11 |  |
|  | ♀+♂ | 47.21 ± 4.48 | 45.99 ± 6.53 | 45.9 ± 6.65 | 45.81 ± 7.74 | 46.87 ± 6.98 | 48.66 ± 9.44 | P< 0.01 |
| ALP | ♀ | 745.60 ± 258.90 | 482.00 ± 167.40 | 268.50 ± 117.30 | 210.80 ± 92.83 | 192.20 ± 92.90 | 171.00 ± 32.86 |  |
| (U/L) | ♂ | 755.8 ± 229.60 | 535.80 ± 214.40 | 437.60 ± 188.30 | 221.40 ± 169.20 | 155.70 ± 85.94 | 138.70 ± 56.49 |  |
|  |  | P=0.82 | P=0.75 | P < 0.01 | P= 0.82 | P = 0.65 | P=0.86 |  |
|  | ♀+♂ | 749.60 ± 247.50 | 358.60 ± 180.00 | 362.50 ± 192.10 | 214.10 ± 121.70 | 174.30 ± 96.33 | 145.40 ± 53.73 | P< 0.01 |
| r-GT | ♀ | 103.00 ± 27.98 | 87.75 ± 20.94 | 70.02 ± 16.81 | 68.29 ± 17.34 | 65.41 ± 18.78 | 72.10 ± 16.67 |  |
| (U/L) | ♂ | 102.7 0± 27.19 | 95.29 ± 19.73 | 92.06 ± 24.04 | 77.26 ± 22.28 | 77.44 ± 23.77 | 80.94 ± 32.07 |  |
|  |  | P=0.88 | P=0.02 | P< 0.01 | P= 0.01 | P=0.02 | P=0.48 |  |
|  | ♀+♂ | 102.40 ± 28.28 | 81.76 ± 23.67 | 85.34 ± 43.30 | 70.78 ± 19.78 | 71.33 ± 22.13 | 79.12 ± 29.56 | P< 0.01 |
