# Supplementary material for: Hematological and biochemical parameters for Chinese rhesus macaque
Source: PLoS One. 2019 Sep 17;14(9):e0222338. doi: 10.1371/journal.pone.0222338 (PMC6748566; doi:10.1371/journal.pone.0222338)
Supplement: S3 Table — (DOCX) [file pone.0222338.s003.docx]

**S3 Table. Renal function index of rhesus macaques.**

| Parameter  (Unit) | Sex | Infants | Juvenile | Young adults | Adults | Middle | Elderly | P values |
| --- | --- | --- | --- | --- | --- | --- | --- | --- |
| UA | ♀ | 18.20 ± 5.68 | 15.33 ± 6.34 | 21.43 ± 7.05 | 14.73 ± 6.45 | 14.62 ± 5.65 | 11.69 ± 9.33 |  |
| (μmol/L) | ♂ | 15.96 ± 5.06 | 12.14 ± 4.11 | 17.62 ± 8.68 | 13.74 ± 7.82 | 11.68 ± 6.12 | 12.97 ± 8.41 |  |
|  |  | P< 0.01 | P< 0.01 | P< 0.01 | P=0.21 | P< 0.01 | P=0.68 |  |
|  | ♀+♂ | 17.32 ± 5.54 | 19.40 ± 8.18 | 12.40 ± 9.42 | 14.42 ± 6.91 | 14.07 ± 11.73 | 12.70 ± 8.48 | P< 0.01 |
| UREA | ♀ | 6.65 ± 2.37 | 6.37 ± 2.02 | 4.97 ± 2.13 | 6.33 ± 2.11 | 6.39 ± 1.91 | 6.73 ± 3.22 |  |
| (mmol/L) | ♂ | 5.63 ± 2.76 | 6.75 ± 1.91 | 6.45 ± 1.32 | 5.95 ± 2.18 | 6.69 ± 1.94 | 6.67 ± 1.29 |  |
|  |  | P< 0.01 | P=0.09 | P< 0.01 | P=0.09 | P=0.42 | P=0.95 |  |
|  | ♀+♂ | 6.25 ± 2.58 | 5.76 ± 1.89 | 5.95 ± 1.72 | 6.211 ± 2.14 | 6.54 ± 1.92 | 6.68 ± 1.79 | P< 0.01 |
| CREA | ♀ | 64.29 ± 16.81 | 53.98 ± 20.03 | 69.72 ± 15.76 | 75.76 ± 24.18 | 73.70 ± 21.16 | 79.63 ± 26.69 |  |
| (μmol/L) | ♂ | 57.58 ± 20.15 | 59.47 ± 20.82 | 78.91 ± 17.18 | 100.30 ± 23.99 | 108.70 ± 35.78 | 103.60 ± 16.76 |  |
|  |  | P< 0.01 | P= 0.02 | P< 0.01 | P< 0.01 | P< 0.01 | P< 0.01 |  |
|  | ♀+♂ | 61.63 ± 18.48 | 74.62 ± 17.14 | 78.76 ± 22.05 | 83.4 ± 26.64 | 90.91 ± 34.04 | 96.44 ± 26.12 | P< 0.01 |
