# Supplementary material for: Hematological and biochemical parameters for Chinese rhesus macaque
Source: PLoS One. 2019 Sep 17;14(9):e0222338. doi: 10.1371/journal.pone.0222338 (PMC6748566; doi:10.1371/journal.pone.0222338)
Supplement: S4 Table — (DOCX) [file pone.0222338.s004.docx]

**S4 Table. Blood glucose and blood lipid index of rhesus macaques.**

| Parameter  (Unit) | Sex | Infants | Juvenile | Young adults | Adults | Middle | Elderly | P values |
| --- | --- | --- | --- | --- | --- | --- | --- | --- |
| GLU | ♀ | 4.62 ± 1.43 | 5.68 ± 1.64 | 3.98 ± 1.35 | 3.56 ± 1.90 | 3.24 ± 1.97 | 3.76 ± 1.41 |  |
| (mmol/L) | ♂ | 4.73 ± 1.51 | 5.43 ± 2.09 | 4.25 ± 1.30 | 3.72 ± 1.82 | 2.80 ± 1.84 | 3.20 ± 1.72 |  |
|  |  | P=0.65 | P=0.12 | P=0.70 | P=0.43 | P=0.17 | P=0.47 |  |
|  | ♀+♂ | 4.68 ± 1.50 | 4.12 ± 1.33 | 4.20 ± 1.94 | 3.61 ± 1.88 | 3.03 ± 1.91 | 3.32 ± 1.66 | P< 0.01 |
| TC | ♀ | 3.23 ± 0.75 | 3.37 ± 1.163 | 2.89 ± 0.83 | 3.04 ± 0.81 | 3.10 ± 0.89 | 2.45 ± 1.20 |  |
| (mmol/L) | ♂ | 3.32 ± 0.642 | 3.56 ± 3.557 | 3.04 ± 0.77 | 2.83 ± 0.87 | 3.34 ± 0.87 | 3.20 ± 0.87 |  |
|  |  | P=0.31 | P=0.04 | P=0.31 | P=0.02 | P=0.10 | P=0.04 |  |
|  | ♀+♂ | 3.27 ± 0.71 | 2.97 ± 0.80 | 2.82 ± 0.77 | 2.98 ± 0.83 | 3.21 ± 0.89 | 3.05 ± 0.98 | P< 0.01 |
| TG | ♀ | 0.86 ± 0.27 | 1.09 ± 0.46 | 1.00 ± 0.47 | 1.00 ± 0.43 | 0.96 ± 0.40 | 0.77 ± 0.32 |  |
| (mmol/L) | ♂ | 0.96 ± 0.32 | 1.21 ± 0.56 | 0.99 ± 0.44 | 0.83 ± 0.52 | 0.88 ± 0.45 | 0.99 ± 0.49 |  |
|  |  | P= 0.02 | P=0.01 | P=0.51 | P< 0.01 | P=0.31 | P=0.21 |  |
|  | ♀+♂ | 0.90 ± 0.29 | 1.00 ± 0.46 | 0.98 ± 0.43 | 0.95 ± 0.47 | 0.92 ± 0.42 | 0.95 ± 0.47 | P< 0.01 |
| HDL-C | ♀ | 1.56 ± 1.20 | 1.44 ± 0.42 | 1.55 ± 0.46 | 1.64 ± 0.47 | 1.71 ± 0.48 | 1.89 ± 0.41 |  |
| (mmol/L) | ♂ | 1.40 ± 0.86 | 1.59 ± 0.85 | 1.55 ± 0.48 | 1.57 ± 0.45 | 1.56 ± 0.51 | 1.78 ± 0.51 |  |
|  |  | P=0.18 | P=0.24 | P< 0.01 | P=0.56 | P=0.46 | P=0.81 |  |
|  | ♀+♂ | 1.50 ± 1.08 | 1.55 ± 0.47 | 2.13 ± 2.00 | 1.62 ± 0.46 | 1.64 ± 0.50 | 1.80 ± 0.49 | P< 0.01 |
| LDL-C | ♀ | 5.74 ± 3.17 | 3.14 ± 2.04 | 3.59 ± 2.34 | 2.21 ± 2.13 | 2.42 ± 2.34 | 1.91 ± 0.82 |  |
| (mmol/L) | ♂ | 4.53 ± 3.63 | 2.80 ± 1.79 | 4.26 ± 2.54 | 3.04 ± 2.75 | 3.05 ± 2.62 | 3.60 ± 3.09 |  |
|  |  | P=0.52 | P=0.07 | P=0.53 | P=0.66 | P=0.84 | P=0.82 |  |
|  | ♀+♂ | 5.29 ± 3.44 | 3.95 ± 2.47 | 4.24 ± 2.94 | 2.47 ± 2.37 | 2.73 ± 2.49 | 3.26 ± 2.85 | P< 0.01 |
