# Supplementary material for: Hematological and biochemical parameters for Chinese rhesus macaque
Source: PLoS One. 2019 Sep 17;14(9):e0222338. doi: 10.1371/journal.pone.0222338 (PMC6748566; doi:10.1371/journal.pone.0222338)
Supplement: S5 Table — (DOCX) [file pone.0222338.s005.docx]

**S5 Table. Immunoglobulin and complement index of rhesus macaques.**

| Parameter  (Unit) | Sex | Infants | Juvenile | Young adults | Adults | Middle | Elderly | P values |
| --- | --- | --- | --- | --- | --- | --- | --- | --- |
| C3 | ♀ | 1.19 ± 0.18 | 1.03 ± 0.23 | 1.29 ± 0.28 | 1.44 ± 0.24 | 1.44 ± 0.27 | 1.22 ± 0.39 |  |
| (g/L) | ♂ | 1.16 ± 0.16 | 1.05 ± 0.27 | 1.28 ± 0.27 | 1.29 ± 0.31 | 1.34 ± 0.38 | 1.31 ± 0.25 |  |
|  |  | P=0.20 | P=0.41 | P=0.07 | P< 0.01 | P=0.03 | P=0.37 |  |
|  | ♀+♂ | 1.18 ± 0.17 | 1.28 ± 0.28 | 1.21 ± 0.22 | 1.40 ± 0.27 | 1.40 ± 0.33 | 1.29 ± 0.28 | P< 0.01 |
| C4 | ♀ | 0.25 ± 0.07 | 0.23 ± 0.09 | 0.26 ± 0.12 | 0.24 ± 0.10 | 0.23 ± 0.11 | 0.25 ± 0.06 |  |
| (g/L) | ♂ | 0.24 ± 0.06 | 0.24 ± 0.13 | 0.27 ± 0.10 | 0.22 ± 0.10 | 0.24 ± 0.13 | 0.22 ± 0.10 |  |
|  |  | P=0.77 | P=0.16 | P=0.23 | P=0.22 | P=0.67 | P=0.44 |  |
|  | ♀+♂ | 0.24 ± 0.07 | 0.26 ± 0.11 | 0.26 ± 0.11 | 0.23 ± 0.10 | 0.23 ± 0.12 | 0.23 ± 0.09 | P< 0.01 |
| IgA | ♀ | 0.54 ± 0.25 | 0.77 ± 0.83 | 1.06 ± 0.36 | 1.14 ± 0.39 | 1.30 ± 0.37 | 0.85 ± 0.45 |  |
| (g/L) | ♂ | 0.63 ± 1.05 | 0.93 ± 1.19 | 1.12 ± 0.37 | 1.22 ± 0.41 | 1.35 ± 0.40 | 1.27 ± 0.34 |  |
|  |  | P=0.40 | P=0.16 | P=0.88 | P=0.46 | P=0.82 | P=0.37 |  |
|  | ♀+♂ | 0.58 ± 0.69 | 1.09 ± 0.37 | 1.46 ± 1.80 | 1.16 ± 0.40 | 1.32 ± 0.38 | 1.18 ± 0.40 | P< 0.01 |
| IgG | ♀ | 6.79 ± 1.87 | 7.32 ± 2.75 | 9.56 ± 2.10 | 11.00 ± 2.80 | 11.35 ± 3.32 | 9.10 ± 3.95 |  |
| (g/L) | ♂ | 6.89 ± 2.26 | 7.75 ± 3.22 | 8.76 ± 1.63 | 9.86 ± 4.06 | 11.86 ± 3.51 | 11.41 ± 3.12 |  |
|  |  | P=0.90 | P=0.20 | P=0.06 | P< 0.01 | P=0.20 | P=0.06 |  |
|  | ♀+♂ | 6.86 ± 2.08 | 9.14 ± 1.90 | 8.54 ± 2.99 | 10.68 ± 3.31 | 11.68 ± 3.52 | 10.93 ± 3.37 | P< 0.01 |
| IgM | ♀ | 0.62 ± 0.30 | 0.77 ± 0.44 | 1.25 ± 0.48 | 1.08 ± 0.55 | 1.03 ± 0.64 | 0.83 ± 0.39 |  |
| (g/L) | ♂ | 0.60 ± 0.30 | 0.82 ± 0.50 | 0.88 ± 0.38 | 0.94 ± 0.46 | 0.94 ± 0.51 | 1.17 ± 0.68 |  |
|  |  | P=0.62 | P=0.42 | P= 0.08 | P= 0.02 | P=0.35 | P=0.16 |  |
|  | ♀+♂ | 0.61 ± 0.30 | 1.05 ± 0.46 | 1.09 ± 0.84 | 1.04 ± 0.53 | 0.99 ± 0.58 | 1.10 ± 0.64 | P< 0.01 |
