# Supplementary material for: Hematological and biochemical parameters for Chinese rhesus macaque
Source: PLoS One. 2019 Sep 17;14(9):e0222338. doi: 10.1371/journal.pone.0222338 (PMC6748566; doi:10.1371/journal.pone.0222338)
Supplement: S6 Table — (DOCX) [file pone.0222338.s006.docx]

**S6 Table. Ion/Electrolyte indexes of rhesus macaques.**

| Parameter  (Unit) | Sex | Infants | Juvenile | Young adults | Adults | Middle | Elderly | P values |
| --- | --- | --- | --- | --- | --- | --- | --- | --- |
| Ca | ♀ | 2.73 ± 0.44 | 2.56 ± 0.28 | 2.63 ± 0.30 | 2.47 ± 0.28 | 2.52 ± 0.33 | 2.79 ± 0.26 |  |
| (mmol/L) | ♂ | 2.71 ± 0.35 | 2.52 ± 0.29 | 2.62 ± 0.38 | 2.62 ± 0.48 | 2.56 ± 0.48 | 2.73 ± 0.39 |  |
|  |  | P=0.06 | P=0.04 | P=0.18 | P< 0.01 | P=0.18 | P=0.74 |  |
|  | ♀+♂ | 2.72 ± 0.40 | 2.62 ± 0.34 | 2.60 ± 0.41 | 2.52 ± 0.36 | 2.54 ± 0.40 | 2.74 ± 0.36 | P< 0.01 |
| Mg | ♀ | 1.03 ± 0.25 | 0.93 ± 0.12 | 0.97 ± 0.14 | 0.97 ± 0.21 | 0.92 ± 0.16 | 1.17 ± 0.38 |  |
| (mmol/L) | ♂ | 1.03 ± 0.15 | 0.94 ± 0.13 | 0.97 ± 0.14 | 0.96 ± 0.16 | 0.95 ± 0.25 | 1.01 ± 0.24 |  |
|  |  | P=0.66 | P=0.74 | P=0.26 | P=0.65 | P=0.53 | P=0.15 |  |
|  | ♀+♂ | 1.03 ± 0.22 | 0.97 ± 0.14 | 0.95 ± 0.17 | 0.96 ± 0.20 | 0.93 ± 0.21 | 1.04 ± 0.28 | P< 0.01 |
| Fe | ♀ | 21.24 ± 7.87 | 24.67 ± 7.06 | 24.50 ± 7.63 | 26.21 ± 7.33 | 22.85 ± 6.83 | 27.09 ± 6.22 |  |
| (μmol/L) | ♂ | 21.93 ± 6.47 | 24.31 ± 6.84 | 27.36 ± 7.84 | 29.95 ± 19.78 | 26.96 ± 11.61 | 27.88 ± 10.84 |  |
|  |  | P=0.15 | P=0.65 | P=0.11 | P=0.29 | P=0.05 | P=0.85 |  |
|  | ♀+♂ | 21.58 ± 7.78 | 26.02 ± 7.87 | 24.87 ± 7.56 | 26.89 ± 9.25 | 24.88 ± 9.67 | 27.72 ± 9.99 | P< 0.01 |
